# Conservative management of airway tear as a complication of silicone endobronchial stenting in bronchomalacia secondary to endobronchial tuberculosis

**DOI:** 10.1002/rcr2.684

**Published:** 2020-11-05

**Authors:** Nai‐Chien Huan, Khai Lip Ng, Mona Zaria Nasaruddin, Noorul Afidza Muhammad, Ummi Nadira Daut, Jamalul Azizi Abdul Rahaman

**Affiliations:** ^1^ Department of Pulmonology Serdang Hospital Kajang Malaysia; ^2^ Department of Medicine Universiti Putra Malaysia Kajang Malaysia

**Keywords:** Bronchomalacia, conservative, silicone stent, tuberculosis

## Abstract

Tracheobronchial stenosis due to tuberculosis (TSTB) is a potentially debilitating complication of endobronchial tuberculosis (EBTB). Endobronchial interventions including silicone stent insertion is an acceptable approach to improve quality of life among patients with TSTB. However, little is known about the optimal management strategy for patients with bronchomalacia secondary to EBTB (B‐EBTB) and whether stent‐related complication rates are higher among this group of patients. Herein, we report two patients with B‐EBTB who unfortunately developed bronchial tear related to silicone endobronchial stenting. Both patients were successfully managed conservatively without the need for emergency open surgery. We hypothesize that endobronchial intervention might be more beneficial for patients with pure TSTB and might be riskier in cases of bronchomalacia with reduced airway thickness and loss of airway cartilaginous support. More future studies are needed to bridge the current gap in knowledge regarding the optimal management and role of endobronchial interventions among patients with B‐EBTB.

## Graphical abstract

We report two patients, both with bronchomalacia due to endobronchial tuberculosis (EBTB), who unfortunately developed bronchial tear related to silicone endobronchial stenting. Both patients were successfully managed conservatively without needing emergency open surgery. This case report highlights the current gap in knowledge regarding (1) optimal management and role of silicone airway stents among patients with bronchomalacia secondary to EBTB (B‐EBTB) and (2) whether stent‐related complication rates are higher among those with B‐EBTB.

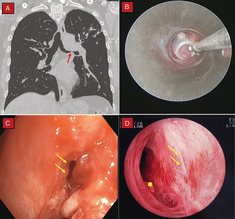

## Introduction

Endobronchial tuberculosis (EBTB), defined as tuberculous infection of the tracheobronchial tree with microbial and/or histopathological evidence, has a variable clinical course and response to treatment [1]. Some patients suffer from debilitating symptoms, necessitating airway intervention such as balloon dilatation, luminal resection, and airway stenting [2, 3, 4, 5, 6, 7, 8]. Silicone airway stent insertion is an acceptable approach to attain meaningful symptomatic relief among patients with tracheobronchial stenosis due to tuberculosis (TSTB) [8]. However, recent evidence pointed that patients with bronchomalacia secondary to EBTB (B‐EBTB) suffer from higher rates of recurrence of symptoms despite airway interventions [9]. Moreover, there is a significant gap in knowledge on whether stent‐related complication rates are higher among patients with B‐EBTB. Herein, we describe two cases of conservative management of bronchial tear related to silicone endobronchial stenting among patients with B‐EBTB.

## Case Report

### Case 1

**Figure 1:**
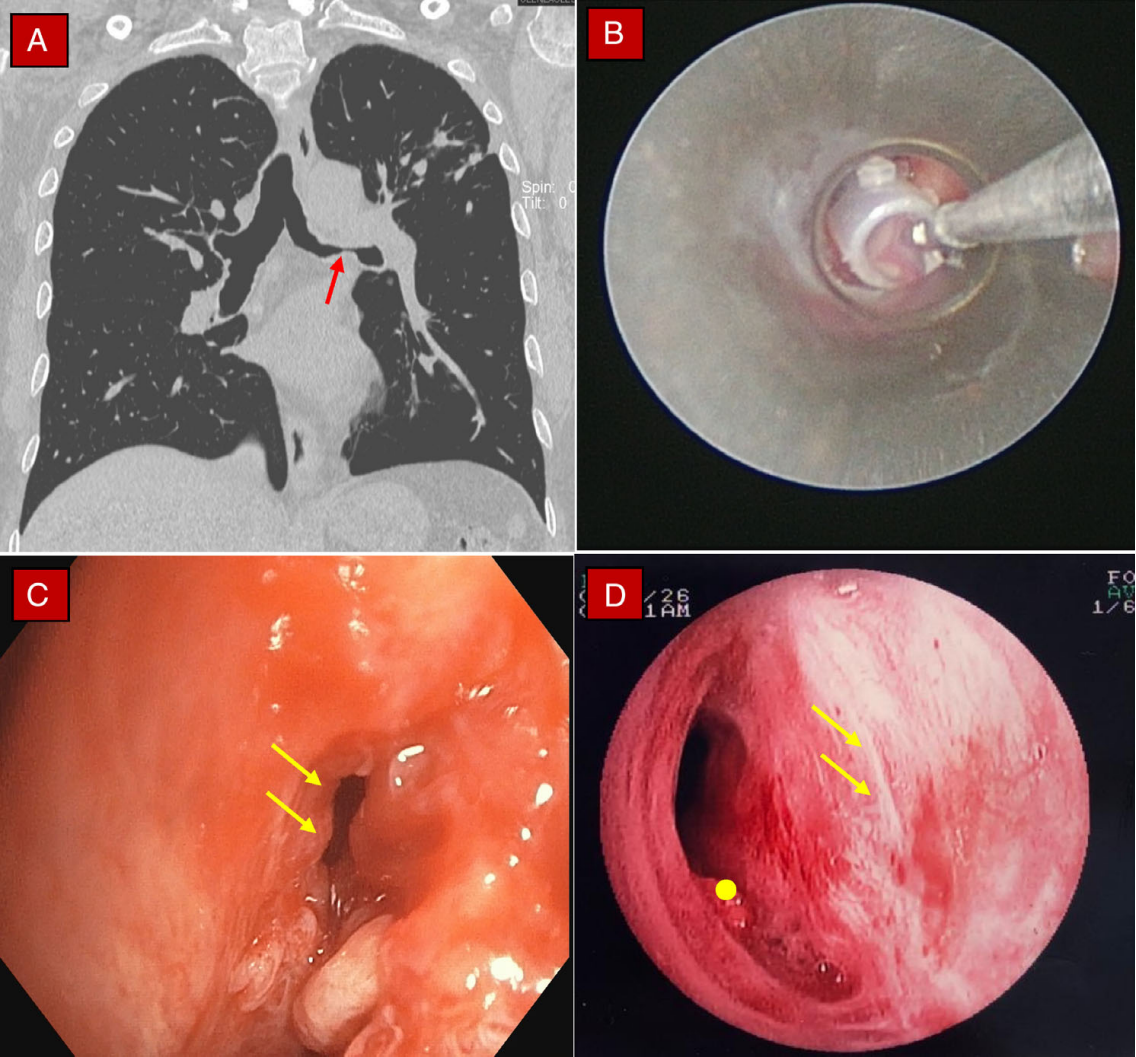
Computed tomography (CT) and serial bronchoscopy images. (A) CT demonstrating narrowing of the left main bronchus due to bronchomalacia (red arrow). (B) Adjusting the position of silicone stent deployed within the bronchomalacic segment by using grasping forceps inserted via rigid bronchoscope. (C) An approximately 2‐cm airway tear at the medial wall of bronchomalacic segment (yellow arrows). (D) Repeated flexible bronchoscopy one month later showing complete healing of bronchial tear (yellow arrows); persistent bronchomalacia marked with yellow circle.

A 57‐year‐old gentleman with a history of pulmonary tuberculosis two years ago was electively admitted for rigid bronchoscopy, balloon dilatation, and silicone airway stent insertion at the left main bronchus. He initially presented with a one‐year history of progressive dyspnoea (scale 3, moderate at rest and scale 5, severe on exertion based on the Modified Borg 1–10 Dyspnoea Scale) without chronic cough or other constitutional symptoms. Computed tomography (CT) scan demonstrated narrowing of the left mainstem bronchi (Fig. 1A). His blood gas results were within normal ranges while three sputum samples for acid‐fast bacilli were negative. Bronchoscopy showed stenosis and marked bronchomalacia of the left main bronchus. The diseased airway segment was 2.5 cm in length while airways distal to the bronchomalacic segment were patent. Careful balloon dilatation of the left main bronchus was done followed by deployment of a bronchial silicone stent (10 mm in diameter, 35 mm in length; NOVATECH® GSS™ BD, Novatech, France) (Fig. 1B). Unfortunately, we noticed a tear of approximately 2 cm in length at the bronchomalacic segment during attempts to correctly position the silicone stent by using rigid grasping forceps (Fig. 1C). The procedure was abandoned immediately and the stent was removed. Post intervention, our patient was kept intubated and sedated and was sent to an intensive care unit for close observation. Urgent CT showed collapse of the left lung but without significant pneumomediastinum or subcutaneous emphysema. Repeated bedside flexible bronchoscopy on the next day revealed thick secretions with evidence of healing at the site of initial airway tear. A multidisciplinary team discussion involving pulmonologists, cardiothoracic surgeons, and patient's family members was conducted to discuss on the best mode of treatment for the patient, that is, conservative management versus open thoracotomy with airway repair. We finally opted for conservative treatment as he remained stable without evidence of clinical deterioration. He was successfully extubated after two days and was discharged well. Repeated bronchoscopy a month later showed complete healing of the airway tear (Fig. 1D).

### Case 2

**Figure 2:**
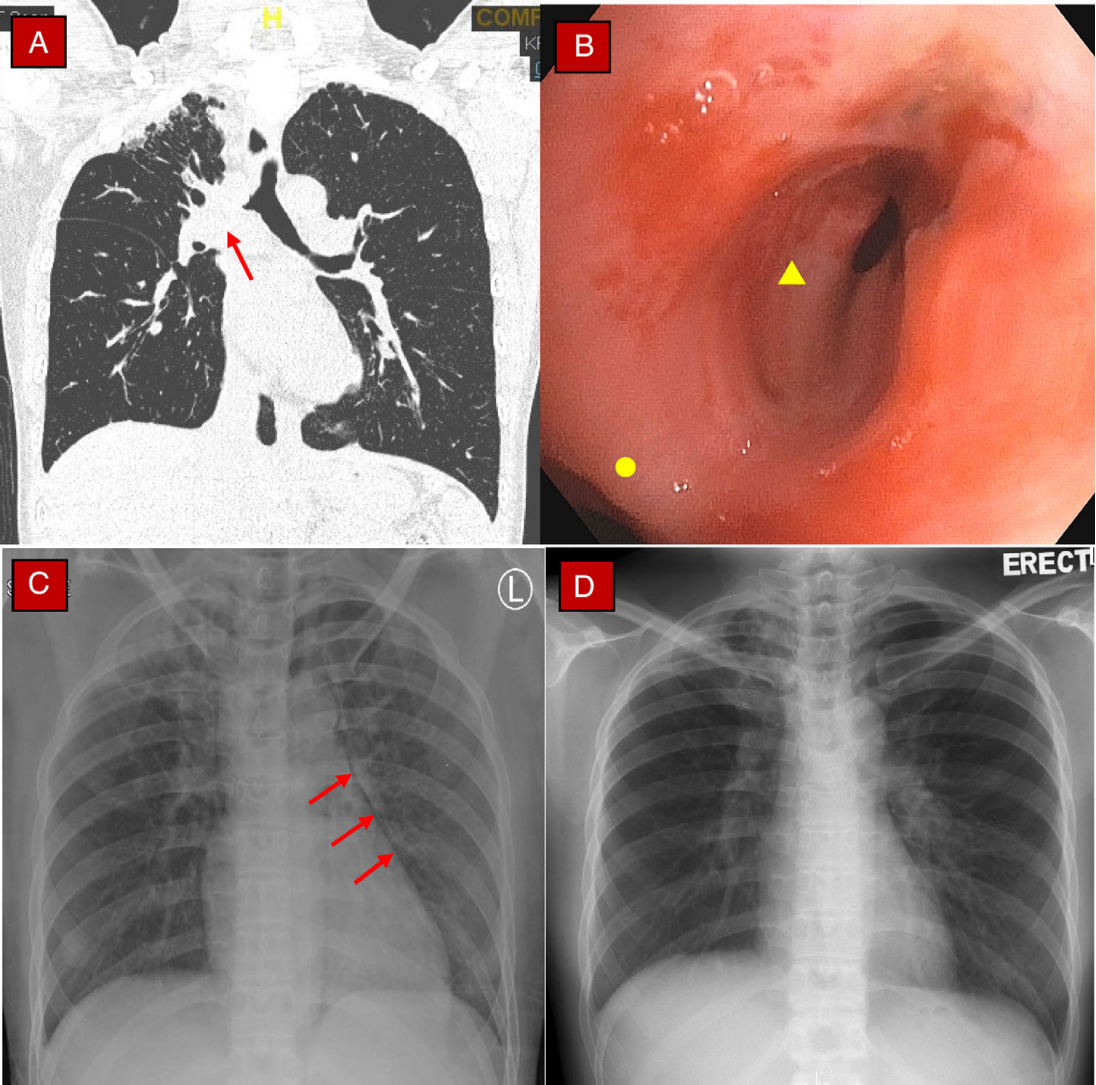
Computed tomography (CT), bronchoscopy image, and serial chest radiograph. (A) CT thorax demonstrating narrowing of the right main bronchus due to bronchomalacia (red arrow). (B) Bronchoscopy showing bronchomalacia of the right main bronchus (yellow triangle); main carina marked with yellow circle. (C) Chest radiograph done immediately post procedure revealing pneumomediastinum (red arrows). (D) Repeated chest radiograph 24 h later showing resolution of pneumomediastinum.

A 38‐year‐old lady with a previous history of pulmonary tuberculosis more than 10 years ago was investigated for a three‐year history of progressively worsening shortness of breath (scale 3, moderate at rest and scale 4, somewhat severe on exertion based on the Modified Borg 1–10 Dyspnoea Scale). CT demonstrated a narrowed airway segment at the right main bronchus (Fig. 2A). Flexible bronchoscopy confirmed the presence of a narrowed and malacic right main bronchus with an opening diameter of approximately 3 mm (Fig. 2B). Blood gases were normal while three consecutive sputum samples for acid‐fast bacilli were negative. She underwent rigid bronchoscopy under general anaesthesia for balloon dilatation of the malacic airway segment. Her right upper lobe bronchus opening was completely obliterated but her right middle and lower lobe segmental bronchi were patent. Post procedure, she reported dramatic improvements in symptoms which lasted for just two weeks. Repeated bronchoscopy showed recurrence of bronchomalacia with a length of approximately 2.5 cm. A decision was made to repeat balloon dilatation together with silicone stent insertion at the right main bronchus. After successful balloon dilatation, an attempt to deploy a straight silicone bronchial stent (10 mm in diameter, 35 mm in length; Stening® Bronchial stent, Animus Beyford Trading, Spain) was unsuccessful due to airway laceration (approximately 0.5 cm in length) at the posterior wall immediately adjacent to the bronchomalacic segment during attempts to adjust the position of silicone stent at the diseased airway segment. The procedure was abandoned and the stent was removed immediately. Repeated chest radiograph post procedure showed signs of pneumomediastinum (Fig. 2C), but we managed to extubate her on the same day of procedure. There was no subcutaneous emphysema noted. Again, a multidisciplinary team discussion was conducted and a decision was made for conservative management (not for urgent thoracotomy and airway repair) as she had remained stable without evidence of clinical deterioration. Repeated chest radiograph on the next day revealed resolution of pneumomediastinum and she was discharged two days later (Fig. 2D).

## Discussion

Chung and Lee classified EBTB into seven subtypes based on bronchoscopic appearances, namely: non‐specific bronchitic, granular, oedematous‐hyperaemic, caseating, ulcerative, tumorous, and fibro‐stenotic [10]. This classification can be regarded as a continuum of EBTB infection: from an early stage (non‐specific bronchitic and oedematous‐erythematous changes) to an intermediate stage characterized by necrosis (seen as airway ulcers or caseous materials) to a late fibrotic healing state (airway fibro‐stenosis). TSTB is a potentially debilitating complication of EBTB as it may irreversibly impair lung physiology resulting in respiratory failure and even death [11].

Various studies have demonstrated that endobronchial intervention, including silicone stenting, could be a useful method for the management of patients with TSTB [2, 3, 4, 5, 6, 7, 8, 9]. Endoscopic intervention is helpful in disease control, relieving symptoms such as dyspnoea, and in improving lung function [9]. However, no study has specifically addressed the best treatment strategy in patients with B‐EBTB. Lee et al. reported that patients with bronchomalacia were 17 times more likely (odds ratio (OR): 17.179, *P* < 0.001) to suffer from recurrent symptoms after airway intervention compared to those with pure TSTB without bronchomalacia [9]. In both of our cases, decisions for airway stenting were made after multidisciplinary meetings involving thoracic surgeons and thoracic radiologists. Both of our patients suffered from long‐segment bronchomalacia (at least 2.5 cm) extending from proximal main bronchi to distal main bronchi, making airway anastomosis post resection difficult during airway surgery. To the best of our knowledge, there were no prior reports addressing airway complications related to endobronchial stenting in patients with B‐EBTB.

In our report, attempts to adjust the position of silicone stent after deployment at the airway segment with bronchomalacia have resulted in bronchial laceration. Chapron et al. described a patient with bronchomalacia due to relapsing polychondritis who developed bronchial rupture during silicone stent insertion [12]. That patient required emergency surgical bronchial repair and subsequent left pneumonectomy. Both of our patients, however, were fortunately successfully managed conservatively without requiring emergency airway surgery or pneumonectomy. Nevertheless, it is worth mentioning that both of our patients suffered from airway tears of not more than 2 cm in length and that procedures were abandoned immediately upon discovery of airway injuries. We believe that urgent imaging and close monitoring in controlled environments with immediate cardiothoracic surgery consult are logical initial approaches for patients who developed such complications. Urgent airway surgery or pneumonectomy should be considered when conservative measures fail, for example, worsening pneumomediastinum, increasing ventilatory requirements, or haemodynamic instability.

B‐EBTB is characterized by airway collapse during exhalation due to destruction of cartilage and decreased airway wall thickness. During endobronchial intervention, the risks of potentially serious complications related to laceration of fragile and collapsed bronchi are probably increased compared to patients without bronchomalacia. Besides, as mentioned previously, recent data suggested that patients with bronchomalacia suffer from higher rates of symptom recurrence despite airway interventions including endobronchial stents [9]. In light of this, we hypothesize that endobronchial intervention might be more beneficial for patients with pure TSTB and might be riskier in cases of bronchomalacia with reduced airway thickness and loss of airway cartilaginous support. More studies are needed to identify the best treatment strategy and the role of endobronchial intervention (vs. airway surgery) among patients with B‐EBTB. We hope that future advances in three‐dimensional printed or customized individualized airway stents will help to resolve this problem.

In conclusion, in the absence of guidelines, B‐EBTB remains a challenging clinical entity to manage. Endobronchial interventions for B‐EBTB appear to be less effective coupled with potential for serious complications including airway tear. In an event of airway tear, initial conservative approach might be appropriate with intense monitoring and proper surgical consult. We hope that more studies in the future can aid to bridge the current gap in knowledge regarding the optimal management and role of endobronchial interventions among this group of patients.

### Disclosure Statement

Appropriate written informed consent was obtained for publication of this case report and accompanying images.

## References

[1] Chan HS , Sun A , and Hoheisel GB . 1990 Endobronchial tuberculosis – is corticosteroid treatment useful? – a report of 8 cases and review of the literature. Postgrad. Med. J. 66:822–826. 2099420 10.1136/pgmj.66.780.822 PMC2429702

[2] Yaguchi D , Kimura H , Inoue N , et al. 2019 Tuberculous bronchial stenosis treated with balloon dilatation. QJM 112:539–540. 30605545 10.1093/qjmed/hcy307

[3] Fang Y , You X , Sha W , et al. 2016 Bronchoscopic balloon dilatation for tuberculosis‐associated tracheal stenosis: a two case report and a literature review. J. Cardiothorac. Surg. 11:21. 26825956 10.1186/s13019-016-0417-z PMC4731900

[4] Watanabe Y , Murakami S , Oda M , et al. 1997 Treatment of bronchial stricture due to endobronchial tuberculosis. World J. Surg. 21:480–487. 9204734 10.1007/pl00012273

[5] Nomori H , Horio H , and Suemasu K . 2000 Granulation stenosis caused by a Dumon stent placed for endobronchial tuberculous stenosis. Surg. Laparosc. Endosc. Percutan. Tech. 10:41–43. 10872526

[6] Iwamoto Y , Miyazawa T , Kurimoto N , et al. 2004 Interventional bronchoscopy in the management of airway stenosis due to tracheobronchial tuberculosis. Chest 126:1344–1352. 15486402 10.1378/chest.126.4.1344

[7] Low SY , Hsu A , and Eng P . 2004 Interventional bronchoscopy for tuberculous tracheobronchial stenosis. Eur. Respir. J. 24:345–347. 15358688 10.1183/09031936.04.00003604

[8] Ryu YJ , Kim H , Yu CM , et al. 2006 Use of silicone stent for the management of post tuberculosis tracheobronchial stenosis. Eur. Respir. J. 28:1029–1035. 16971412 10.1183/09031936.00020906

[9] Lee KCH , Tan S , Goh JK , et al. 2020 Long‐term outcomes of tracheobronchial stenosis due to tuberculosis (TSTB) in symptomatic patients: airway intervention vs. conservative management. J. Thorac. Dis. 12(7):3640–3650. 32802443 10.21037/JTD-20-670 PMC7399429

[10] Chung HS , and Lee JS . 2000 Bronchoscopic assessment of the evolution of endobronchial tuberculosis. Chest 117(2):385–392. 10669679 10.1378/chest.117.2.385

[11] Kashyap S , and Solanki A . 2014 Challenges in endobronchial tuberculosis: from diagnosis to management. Pulm. Med. 2014:594806. 25197570 10.1155/2014/594806 PMC4147266

[12] Chapron J , Wermert D , Le Pimpec‐Barthes F , et al. 2012 Bronchial rupture related to endobronchial stenting in relapsing polychondritis. Eur. Respir. Rev. 21:367–369. 23204127 10.1183/09059180.00000612 PMC9487227

